# Expression of transforming growth factor-β (TGF-β) in chronic idiopathic cough

**DOI:** 10.1186/1465-9921-10-40

**Published:** 2009-05-22

**Authors:** Shaoping Xie, Patricia Macedo, Mark Hew, Christina Nassenstein, Kang-Yun Lee, Kian Fan Chung

**Affiliations:** 1 Airway Disease Section, National Heart & Lung Institute, Imperial College & Royal Brompton Hospital, London SW3 6LY, UK; 2 Fraunhofer Institute of Toxicology and Experimental Medicine, Hannover, Germany

## Abstract

In patients with chronic idiopathic cough, there is a chronic inflammatory response together with evidence of airway wall remodelling and an increase in airway epithelial nerves expressing TRPV-1. We hypothesised that these changes could result from an increase in growth factors such as TGFβ and neurotrophins.

We recruited 13 patients with persistent non-asthmatic cough despite specific treatment of associated primary cause(s), or without associated primary cause, and 19 normal non-coughing volunteers without cough as controls, who underwent fiberoptic bronchoscopy with bronchoalveolar lavage (BAL) and bronchial biopsies.

There was a significant increase in the levels of TGFβ in BAL fluid, but not of nerve growth factor(NGF) and brain-derived nerve growth factor(BDNF) compared to normal volunteers. Levels of TFGβ gene and protein expression were assessed in bronchial biopsies. mRNA expression for TGFβ was observed in laser-captured airway smooth muscle and epithelial cells, and protein expression by immunohistochemistry was increased in ASM cells in chronic cough patients, associated with an increase in nuclear expression of the transcription factor, smad 2/3. Subbasement membrane thickness was significantly higher in cough patients compared to normal subjects and there was a positive correlation between TGF-β levels in BAL and basement membrane thickening.

TGFβ in the airways may be important in the airway remodelling changes observed in chronic idiopathic cough patients, that could in turn lead to activation of the cough reflex.

## Background

Chronic cough is a common clinical problem [1,2]. Asthma, postnasal drip or rhino-sinusitis, and gastro-oesophageal reflux have been recognized as being the most common causes of chronic cough [2,3]. In some patients, no cause can be identified despite thorough investigations and empiric treatment [4-6], a group recently denoted as 'idiopathic'. Patients with chronic cough very often demonstrate an increased tussive response to inhalation of tussive agents such as capsaicin indicates that there is a sensitisation of the cough reflex [7]. Both peripheral and central causes of this sensitisation have been put forward[8,9]; however, changes observed in the airways of patients with chronic cough indicate that peripheral changes could be involved in the sensitisation of the cough reflex. Thus, there is an increase in mediator expression as measured by increased levels of histamine in bronchoalveolar lavage fluid, and levels of cys-leukotrienes, leukotriene B4, myeloperoxidase and TNFα in induced sputum samples from patients with persistent cough [10]. Examination of bronchial biopsies from non-asthmatic chronic cough patients reveal an increase in mast cells in the submucosa, with also marked changes in airway wall remodelling such as subepithelial fibrosis, goblet cell hyperplasia and blood vessels, similar to that observed in patients with asthma, together with an increase in airway smooth muscle cells [11]. Perhaps of greater relevance to the enhanced cough reflex are abnormalities in the epithelial nerve profiles which could represent cough receptors. Although there are no increases in nerve profiles, the expression of the neuropeptide, calcitonin gene-related peptide (CGRP), and of the ion channel, transient receptor potential vanniloid 1 (TRPV1), has been reported to be increased in these epithelial nerves [12,13].

To explore further the role of airway wall remodelling and of peripheral neural plasticity in chronic cough, we have measured in bronchoalveolar lavage fluid the levels of growth factors, such as transforming growth factor-β (TGFβ), which may be involved in subepithelial fibrosis [14], and of the neurotrophins such as brain-derived neurotrophin (BDNF) which may elicit sensitisation of nociceptors [15], and angiogenesis and microvascular remodelling [16]. We also examined the expression of TGFβ in airways submucosa of chronic idiopathic cough patients.

## Methods

### Subjects

We studied patients with chronic cough of at least 8 weeks' duration referred to our cough clinic and excluded patients who had a diagnosis of asthma as a cause of their cough (Table 1). As a control group, we recruited normal volunteers through local advertisement; these normal volunteers had no previous history of cough or asthma, and were not suffering from any intercurrent illness.

**Table 1:** Patient characteristics

	Normals	Chronic cough
Number	13	20

Gender (Male/Female)	9:4	4:16**

Age (years)	19.9 ± 0.4	55.4 ± 2.5**

Smoking status (n)		

never	12	11

ex-smoker	1	9

Pack-years	7	13.1 ± 3.5

GORD (n)	NA	10
Postnasal drip (n)	NA	7
Neither (n)	NA	5

Atopy (%)	31	26

Capsaicin (log_10 _C5)	ND	0.53 ± 0.14

FEV_1 _(% predicted)	96.0 ± 3.3	99.0 ± 2.3

FVC (% predicted)	100.5 ± 3.3	96.8 ± 4.6

*Bronchoalveolar lavage*		

% macrophages	97.6 ± 0.5	90.1 ± 2.6**

% neutrophils	1.5 ± 0.4	6.7 ± 2.6*

% lymphocytes	0.7 ± 0.2	3.0 ± 0.8**

% eosinophils	0.3 ± 0.1	0.2 ± 0.1

FEV_1_: Forced expiratory volume in one second; FVC: Forced vital capacity; GORD: gastro-oesophageal reflux disease; NA: Not applicable; ND: Not done. Data shown as mean ± SEM. * p < 0.05; **p < 0.01.

Patients with chronic cough underwent diagnostic evaluation that included chest radiograph, pulmonary function test, methacholine challenge, 24-hour oesophageal pH-monitoring, and chest and sinus computed tomography[1]. Patients with airway hyperresponsiveness (provocative concentration of methacholine that induced a > 20% decrease of forced expiratory flow in 1 second (FEV_1_) [PC_20_- FEV_1_] < 4 mg/ml), diurnal variation of peak expiratory flow (> 20%), or > 15% increase of FEV_1 _after β-agonist, and also response of coughing to inhaled bronchodilator and corticosteroid therapy were diagnosed as having asthma responsible for chronic cough, and they were excluded from the study. The patients with chronic cough recruited to this study had a PC_20 _FEV_1 _> 8 mg/ml. Chronic cough due to gastro-oesophageal reflux was diagnosed by 24-hour oesophageal pH-monitoring and efficacy of 12-week course of proton-pump inhibitor, and dietary changes. Chronic cough was attributed to post-nasal drip/rhinosinusitis when symptoms and objective diagnosis of postnasal drip and/or rhinosinusitis were present and nasal corticosteroids and/or nasal anticholinergics were effective against cough. Some patients had no identifiable cause(s) of cough despite additional investigations including bronchoscopy and intensive therapeutic trials for asthma, gastro-esophageal reflux and postnasal drip/rhinosinusitis, and were labelled as 'idiopathic'. Only ex-smokers who have ceased smoking more than 12 months of enrolled were recruited.

The study was approved by the Ethics Committee of our institution and all subjects gave informed consent to participate in the study.

### Capsaicin challenge

As previously described [17], coughs were counted for one minute after single-breath inhalations of 0.9% sodium chloride and capsaicin solutions of increasing concentrations (0.98 to 500 μM). They were generated from a dosimeter (P.K.Morgan Ltd, Gillingham, UK) set at a dosing period of 1 second. This was continued until 5 or more coughs were induced. The concentration that caused 5 or more coughs was recorded (C5) and the data analysed as log_10 _C5.

### Bronchoscopy, bronchoalveolar lavage and bronchial biopsy

Bronchoscopy was performed as previously described [18]. Briefly, subjects were pretreated with intravenous midazolam (5 mg). Oxygen was administered via nasal prongs throughout the procedure. Using local anesthesia with 2% lidocaine to the upper airways and larynx, a fibreoptic bronchoscope (Olympus BF 10, Key-Med, Herts, UK) was passed through the nasal passages into the trachea. Warmed 0.9% NaCl solution (50 ml × 4) was instilled into the right middle lobe and BAL fluid was retrived by gentle suction. The supernatant was recovered after centrifugation of the fluid and kept at -70C in aliquots of 5 mls until assayed. Washed BAL cells were suspended in culture media and counted on a hemocytometer. Cytospins were stained with DiffQuick stain for differential cell counts. Three to 5 mucosal biopsies were taken from the segmental and subsegmental bronchi of the right lower lobe.

### Measurement of TGF-β1, NGF and BDNF in BAL fluid

The concentrations of TGF-β1, NGF and BDNF in BALFs were measured by ELISA kits according to the manufacturer's instructions (R&D System or Promega for BDNF). For TGF-β1 assay, BALFs were first activated by incubation with 1N HCl for 10 min and neutralized by 1.2 N NaOH/0.5 M *N*-2-hydroxyethylpiperazine-*N'*-ethane sulfonic acid. Activated samples were then transferred to the wells of plates coated with TGF-β1 soluble receptor Type II. For NGF and BDNF assay, plates were coated with anti-human β-NGF or BDNF antibody. 100 μl of BALFs were added to each well. After incubation and thorough washing, specific antibody for each measurement was added to the test wells. TGF-β1, NGF and BDNF were detected using a horseradish peroxidase-based colorimetric assay.

### Laser Capture Microdissection

Human airway biopsies were embedded in Optimum Cutting Temperature compound (OCT) on dry ice and snap-frozen in liquid nitrogen before storage at -80°C. Frozen sections were cut at 6 μm thickness and mounted on Laser Capture Microdissection (LCM) slides (Arcturus, Mountain View, California, US). The slides were immediately stored on dry ice and then at -80°C until used. Sections were fixed in 70% ethanol for 30 seconds, and stained and dehydrated in a series of graded ethanol followed by xylene using HistoGene LCM frozen section staining kit (Arcturus) according to the manufacturer's instruction. Airway smooth muscle (ASM) cells or epithelial cells were captured onto the CapSure HS LCM caps (Arcturus) by a Pixcell II Laser Capture Microdissection System (Arcturus).

### Real-time PCR

Total RNA was extracted by using a PicoPure RNA isolation kit (Arcturus) according to the manufacturer's instructions and was reverse transcribed to cDNA (RoboCycler, Stratagene, USA) using random hexamers and AMV reverse transcriptase (Promega). cDNA was amplified by quantitative real-time polymerase chain reaction (PCR) (Rotor Gene 3000, Corbett Research, Australia) using SYBR Green PCR Master Mix Reagent (Qiagen). The human TGF-β1 forward and reverse primers were 5'-CCCAGCATCTGCAAAGCTC-3' and 5'-GTCAATGTACAGCTGCCGCA-3'. Each primer was used at a concentration of 0.5 μM in each reaction. Cycling conditions were as follows: step 1, 15 min at 95°C; step 2, 20 sec at 94°C; step3, 20 sec at 60°C; step 4, 20 sec at 72°C, with repeat from step 2 to step 4 for 40 times. Data from the reaction were collected and analysed by the complementary computer software (Corbett Research, Australia). Relative quantitations of gene expression were calculated using standard curves and normalized to 18S rRNA in each sample.

### Immunohistochemistry

Immunohistochemistry was performed to detect the protein expression of TGF-β1 in human bronchial tissue sections. Bronchial biopsies were embedded in OCT and stored at -80°C before use. Frozen sections (6 μm) were cut before fixed in cold acetone for 10 min. Sections were incubated in 10% normal horse serum to block non-specific binding, followed by a mouse anti-human TGF-β1 antibody (1 μg/ml, AbCam ab1279) for 1 hour at room temperature. Control slides were performed with normal mouse immunoglobulin. Anti-mouse biotinylated secondary antibody (Vector ABC Kit, Vector Laboratories) was applied to the sections for 1 hour at room temperature, followed by 1.6% hydrogen peroxide to block endogenous peroxidase activity. Sections were incubated with the avidin/biotinylated peroxidase complex for 30 min, followed by chromogenic substrate diaminobenzidine for 3 min, and then counterstained in haematoxylin and mounted on aqueous mounting medium. Immunoreactivity for TGF-β1 was expressed as intensity of staining that was graded from 0 to 4. Slides were read blindly.

### Subbasement membrane thickness

Frozen sections were stained with haematoxylin and eosin. Subbasement membrane thickness was assessed (NIH Image analysis 1.55) by measuring 40 point-to-point repeated measurements at 20 μm intervals per biopsy and the mean thickness calculated as previously reported [19].

### Immunofluorescence and laser scanning confocal microscopy

Immunofluorescence was carried out to detect the nuclear translocation of Smad2/3 in human bronchial biopsy sections. The sections were fixed with 2% paraformaldehyde for 10 min at room temperature before incubating in 5% normal donkey serum to block non-specific binding. Smad2/3 activation was detected using the rabbit polyclonal antibody for Smad2/3 (1:50, Upstate 07–408, Watford, UK) for 1 hour at room temperature. Sections were then incubated with a rhodamine-conjugated donkey anti-rabbit IgG (1:100) for 45 min in the dark and counterstained with DAPI solution. Sections were photographed with a laser scanning confocal microscope.

### Data analysis

Data were analysed by unpaired non-parametric t-test. Results are expressed as mean ± SEM. P < 0.05 was taken as statistically significant.

## Results

Table 1 shows the characteristics of the chronic cough patients and the control volunteers who underwent the fiberoptic bronchoscopic procedure. The control group was significantly younger than the chronic cough group. In the chronic cough group. Ten and 7 patients had an associated diagnosis of gastro-oesophageal reflux and postnasal drip respectively, and in 5, no such associated cause was found.

### TGF-β1, NGF and BDNF levels in BAL fluid

**Figure 1:**
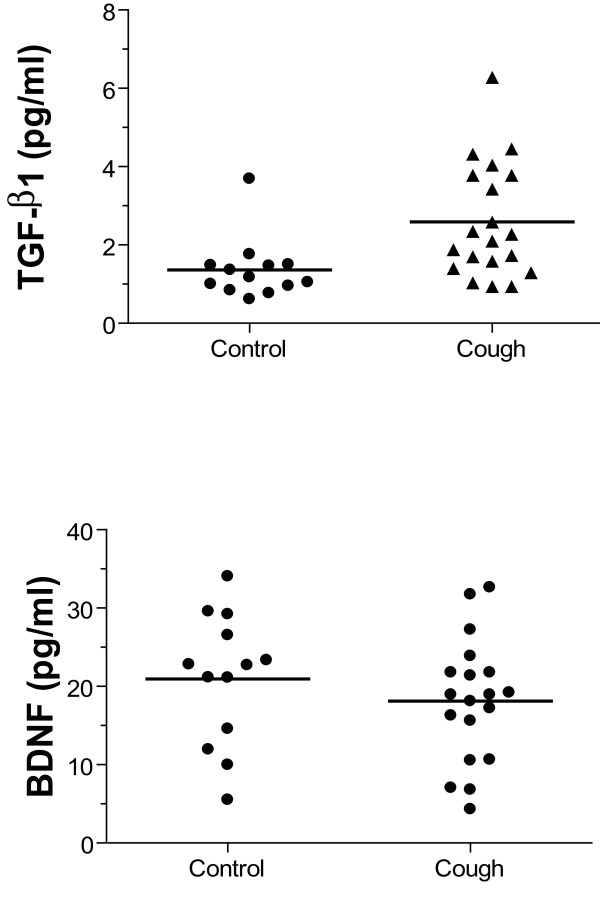
**Activated TGF-β1 (Panel A) and BDNF (Panel B) levels in bronchoalveolar lavage fluid (BALF) from 13 normal subjects and 20 chronic cough patients**. **p < 0.005 compared with normal control. NS: not significant. Horizontal bar shows mean.

BAL levels of TGF-β1 were significantly higher in chronic cough compared to those from non-coughing controls (Figure 1). The mean level was 1.7-fold higher, but in nearly half of the chronic cough patients, the levels were significantly higher.

BDNF levels were not different between the two groups, and levels of NGF were below the limit of detection (Figure 1). There was a negative correlation between the levels of BDNF and those of TGF-β1 in the chronic cough patients (r = -0.67; p < 0.01), indicating that high levels of BDNF were associated with low levels of TGF-β1. There was no correlation between age and the levels of any of these growth factors in BALF. There was no correlation between log C5 and TGFβ1 or BDNF levels in BALF.

### TGF-β1 protein expression in bronchial biopsies

**Figure 2:**
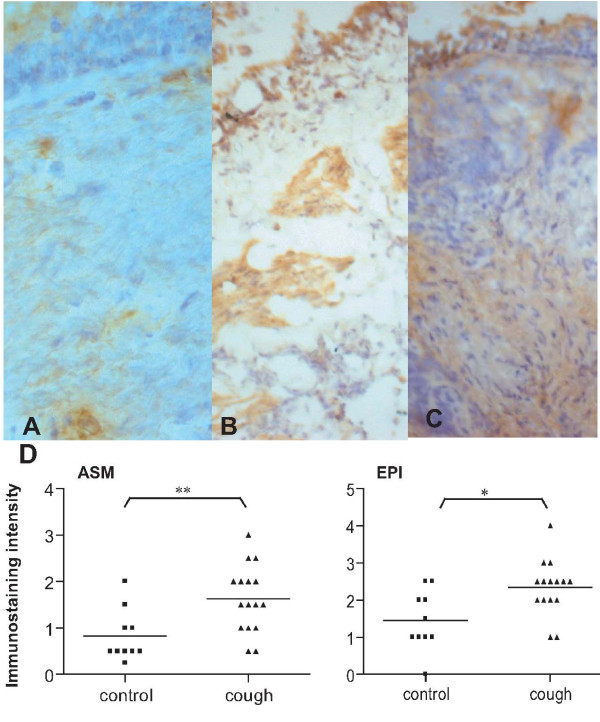
**Expression of TGFβ1 protein in bronchial biopsies**. Examples of TGFβ1 expression in biopsies from normal (Panel A) and from chronic cough patients (Panels B and C) patients. There is increased staining for TGFβ in the airway smooth muscle and epithelial cells in the biopsies from chronic cough patients. Negative control where the primary antibody has been replaced by normal rabbit immunoglobulin does not show any staining (not shown). Magnification is ×400 for Panels A and B, ×200 for Panel C. Panel D. Immunostaining intensity for TGFβ1 (grade 0 to 4) in epithelium (EPI) and in airway smooth muscle (ASM) from 10 normal and 16 cough patients. **p < 0.01, *p < 0.05; horizontal bar shows the mean.

In view of the increase in TGF-β1 levels in BALF from chronic cough patients, we next performed immunohistochemistry in the biopsy samples from 10 normal and 16 cough donors. TGF-β1 expression was enhanced in airway smooth muscle and epithelium of chronic cough patients compared with normal controls (Figure 2). TGF-β immunostaining intensity was higher by 2-fold and 1.6-fold in ASM (p = 0.009) and epithelium (p < 0.02), respectively, of chronic cough patients compared to normal controls (Figure 2B). There was no positive staining in the negative control sections in which the mouse anti-TGF-β1 antibody was replaced by normal mouse immunoglobulin (Figure 2A).

### TGF-β1 mRNA in laser-captured airway smooth muscle and epithelium

**Figure 3:**
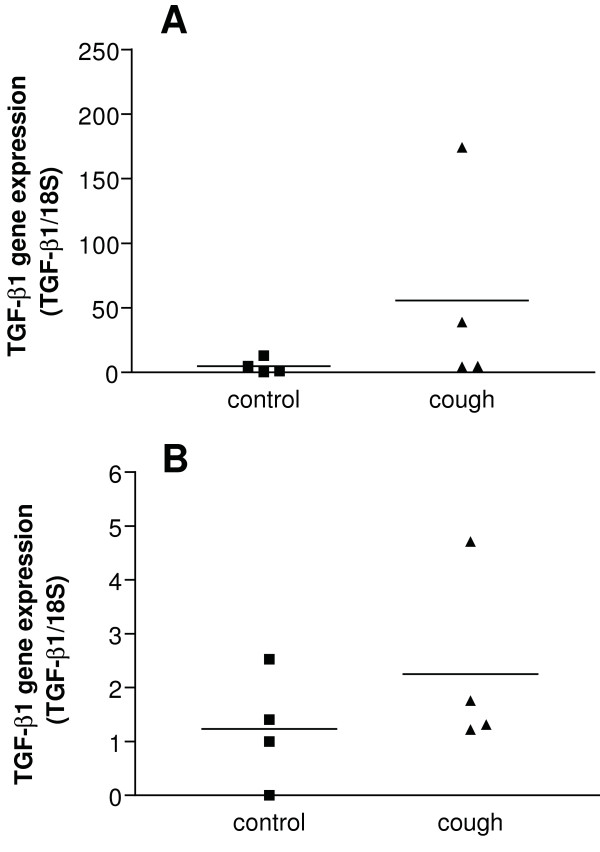
**TGF-β1 mRNA expression in airway smooth muscle (Panel A) and epithelial cells (Panel B) obtained by laser capture microdissection**. TGF-β1 mRNA expression measured by real-time RT-PCR and expressed as a ratio of 18S rRNA is shown for a sample of 4 normal and 4 chronic cough patients. There was no significant difference.

We further examined whether the increased expression of TGF-β protein was related to increased mRNA level in smooth muscle and epithelium of bronchial biopsies from 4 chronic cough patients and 4 controls. These *in-situ *airway smooth muscle and epithelial cells expressed TGF-β1 mRNA, with a trend for a greater level of expression in cells from chronic cough patients but statistical significance was not achieved (Figure 3A &3B).

### Subbasement membrane thickness

**Figure 4:**
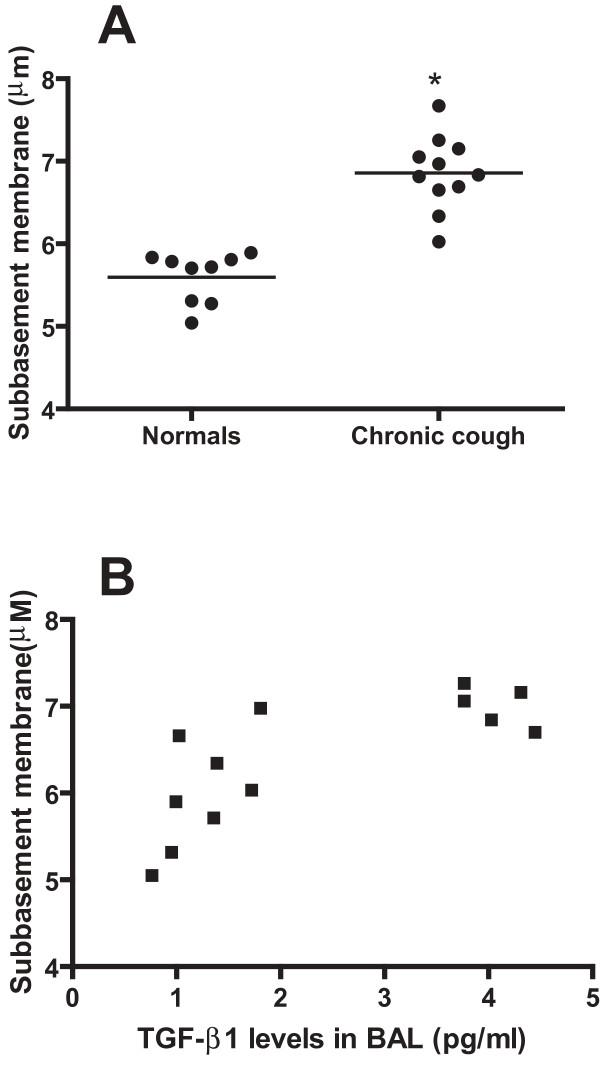
**Subbasement membrane thickness**. Panel A shows increased subbasement membrane thickness in chronic cough patients compared to normal controls(* p < 0.0001). The horizontal bar shows the mean value. Panel B shows that the subbasement membrane thickness correlated with TGF-β levels in bronchoalveolar lavage fluid (r = 0.82, p < 0.001).

Subbasement membrane thickness was significantly increased in chronic cough patients compared to healthy controls (p < 0.0001; Figure 4A) There was a positive correlation between subbasement membrane thickness and TGF-β levels in BAL fluid (n = 13; r = 0.82; p < 0.0006; Figure 4B), but not with the intensity of TGF-β staining in the biopsies.

### Smad2/3 activation in bronchial biopsies

**Figure 5:**
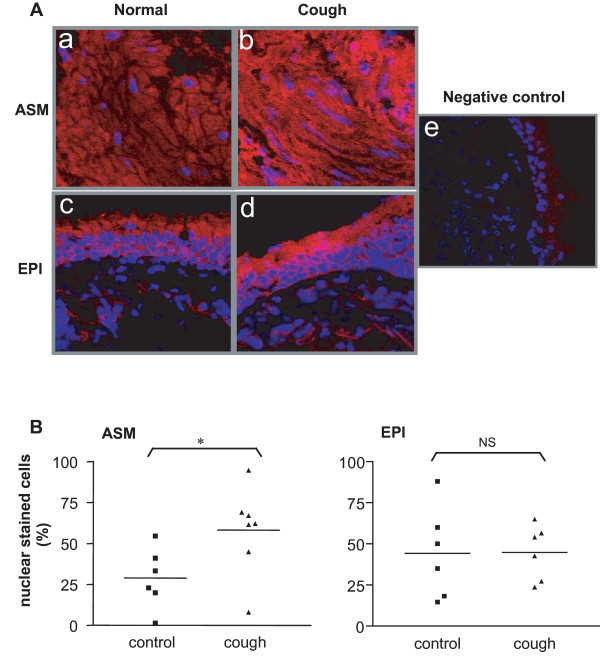
**Smad2/3 expression in bronchial biopsies**. Panel A. Immunofluorescence pictures from confocal microscopy for Smad2/3 activation (red fluorescence) from human bronchial biopsy sections. Nuclei are stained blue with DAPI. For the negative control, the primary antibody was replaced by a normal rabbit immunoglobulin. ASM: airway smooth muscle; EPI: epithelium. Panel B: % of cells with positive nuclear staining for Smad 2/3 in bronchial biopsies from 6 normal and 7 chronic cough patients. *p < 0.05 compared with normal control. NS: not significant.

Because TGF-β induces Smad2/3 phosphorylation and nuclear translocation, we examined for the presence of Smad2/3 activation using immunofluorescence confocal microscopy for nuclear staining. The level of Smad2/3 activation expressed as % of nuclear staining cells was higher in ASM cells of chronic cough patients compared with normal controls (p < 0.05) (Figure 5A &5B). There was no difference of Smad2/3 nuclear staining in airway epithelium between chronic cough patients and normal controls.

## Discussion

We found that TGFβ levels were increased in bronchoalveolar lavage fluid and also in immunohistochemical sections of the bronchial mucosa, particularly expressed in the airway epithelium and airway smooth muscle cells from patients with chronic idiopathic cough compared to normal volunteers. These indicate that there is an increased amount of TGFβ expressed in the airways that could be involved in the airway wall remodelling of chronic cough. This is supported by the findings of a positive correlation between subbasement membrane thickness and TGF-β levels in BAL. In addition, the increased activation of smad 2/3 observed in the bronchial tissues also indicate that TGFβ is may be active. Of particular interest, an increase in TGFβ has also been reported in BAL fluid from asthma patients, but none of the patients with chronic cough had any features of chronic asthma that could be underlying their cough.

An increased expression of TGFβ has also been reported in the airway epithelium and airway smooth muscle cells of patients with asthma [20,21]. We examined for the presence of TGFβ mRNA expression in the airway epithelial cells and airway smooth muscle cells using the technique of laser capture for the first time, allowing us to specifically pinpoint the expression of TGFβ in these selected cells. However, no significant differences in TGFβ gene expression were observed in patients with chronic cough compared to normal volunteers; however, the number of patients in each group was probably too small to be conclusive. However, we can be confident that there is TGFβ gene expression present in the basal state in these airway cells, but cannot be definite as to the differences in expression of TGFβ message. Levels of BDNF in BAL fluid were not increased and levels of NGF were undetectable. This is in agreement with a previous study that measured neurotrophin levels in the supernatants of induced sputum, and found no differences between the chronic coughers and controls [22].

Although the control group of non-coughing volunteers was not balanced in terms of age and gender as compared to the chronic cough patients, it showed no evidence asthma or of chronic airflow obstruction. The difference in TGFβ levels is unlikely to be explained by age or gender differences since there was no correlation between age and TGFβ levels or differences in levels of TGFβ between the male and female gender. This discrepancy occurred as a result of difficulty in recruiting non-smoker controls particularly middle-aged women to undergo fiberoptic bronchoscopy. Nine out of the 20 chronic cough patients were ex-smokers and how this could have influenced the expression of TGFβ in our studies is unclear. A previous study has in fact shown that TGFβ expression is increased in smoking COPD patients compared to smoking non-COPD patients[23]. None of our patients showed evidence of chronic airflow obstruction.

The patients with chronic cough recruited in the present study did not respond to any specific treatment of associated causes such as asthma, gastrooesophageal reflux and postnasal drip. No diagnostic cause of the cough could be determined in all patients. Often, an empirical treatment of the common causes of cough had been given, namely asthma treatments with inhaled corticosteroids, or proton pump inhibitors or nasal corticosteroids. These patients have all been categorised as having an idiopathic cough, in whom we could not find a treatable cause or a cause that is responsive to specific therapies of their cough. This condition of 'idiopathic' cough can range from 7 to 46% of all patients attending cough clinics where a thorough systematic diagnostic work-up is performed[1]. A possible explanation of the cause of idiopathic cough is that the initiating cause of the cough may have disappeared, but its effect in enhancing the cough reflex may be more prolonged. An example would be the transient appearance of an upper respiratory tract virus infection or an exposure to toxic fumes, that results in prolonged damage of the airways mucosa. The cough becomes 'idiopathic' when the primary inciting cause has resolved while cough is persistent. The repetitive mechanical and physical effects of coughing bouts on airway cells could activate the release of various chemical mediators that could enhance chronic cough through inflammatory mechanisms, providing a positive feed-forward system for cough persistence. There may be an induction in the upper airways of inflammation and tissue remodelling induced by various causes associated with cough or by the act of coughing itself that could lead to an enhanced cough reflex, that in turn is responsible for maintaining cough.

Previous studies have reported that mucosal biopsies taken from a group of non-asthmatic patients with chronic dry cough showed evidence of epithelial desquamation and inflammatory cells, particularly lymphocytic inflammation, and also by an increase in submucosal mast cells, but not of neutrophils or eosinophils, with goblet cell hyperplasia, subepithelial fibrosis and increased vascularity [24]. Increased mast cells have been observed also in bronchoalveolar lavage fluid [25] and increased neutrophils in induced sputum [26], with increased concentration of histamine, PGD_2 _and PGE_2_, together with TNFα and IL-8 in induced sputum [27]. These inflammatory changes may not be specific for idiopathic cough because they could represent the sequelae of chronic trauma to the airway wall following repeated episodes of cough. It is also possible that chronic airway wall remodelling may represent the effects of the putative aetiological factor for cough, namely growth factors released that induced the remodelling changes, and also that could change cough receptor sensitivity.

Release of growth factors such as those of the nerve growth factor family may lead to alterations in the phenotype of neural tissues. Nerve growth factor (NGF) may increase the expression of calcitonin gene-related peptide (CGRP) [28] and TRPV-1 [29] in nerves. Elevation of CGRP and TRPV-1 has been reported in airway epithelial nerves in chronic cough [12,13]. However, there is no evidence for an increase in NGF levels in BAL fluid or induced sputum supernatants [21].

Our work supports a new concept regarding the persistence of chronic idiopathic cough through the activation of TGF-β. Airway epithelial cells may produce growth factors such as TGF-β and endothelin and epidermal growth factor (EGF)-like growth factors when subjected to mechanical stress or pressure [30,31]. Therefore, the repetitive mechanical and physical effects of coughing bouts on the airway cells, particularly the airway epithelium, may be responsible for the increased release of TGFβ. This possibility is supported by a recent study that showed that traumatic mechanical stress to the large airways can induce a neutrophilic airway inflammation together with cough reflex hypersensitivity [32]; however, the expression of TGFβ was not examined in this study.

TGFβ has been implicated as a growth factor in the remodelling of the epithelial-mesenchymal trophic unit as it can induce the expression of extracellular matrix components [33]. In addition, TGFβ can induce the proliferation and hypertrophy of airway smooth muscle cells [34,35], and since these cells also express TGFβ, as demonstrated in the current study, potential autocrine effects are also possible. TGFβ can also induce mesenchymal cells such as fibroblasts and airway smooth muscle cells to release chemokines such as IL-8/CXCR8, eotaxin/CCR3 or monocyte chemoattractant protein-1(MCP-1)/CCL2, that may contribute to the cellular inflammatory response [36-38]. These biological effects of TGFβ support a role for TGFβ in chronic cough, at least as a potential explanation for the remodelling and inflammatory changes observed in the airway mucosa of chronic cough patients [11]. However, there is no information as to whether TGFβ can act as a sensitiser of the capsaicin cough reflex that is enhanced in chronic cough. While it is known that TGFβ exists in at least 3 isoforms, only the TGFβ1 isoform has been studied in the current study. Evaluation of other isoforms is important as demonstrated with the increased TGFβ2 isoform in a study of patients with severe asthma [39]

The link between TGFβ and persistent cough is unclear. Could airway wall remodeling in which TGFβ is involved be the basis for the cough? TGFβ is a growth factor involved in airway wall remodelling and whether the fibrotic changes in the airway can alter cough receptor sensitivity is not known. Whether chronic cough leads to airway wall remodelling or airway wall remodelling is a cause of chronic cough is difficult to determine but the concomitance of both mechanisms may form the basis of a positive feedback mechanism for cough persistence.

## Competing interests

The authors declare that they have no competing interests.

## Authors' contributions

SX performed studies on laser-captured cells and the immunostaining, PM & MH performed the fiberoptic bronchoscopies, CN did the assay for neurotrophins, K-YL performed the assay for TGFβ, KFC designed the study and all authors contributed to the writing up.
